# Comparative analysis of early visual processes across presentation modalities: The event-related potential evoked by real-life, virtual reality, and planar objects

**DOI:** 10.3758/s13415-025-01294-0

**Published:** 2025-04-08

**Authors:** Joanna Kisker, Marike Johnsdorf, Merle Sagehorn, Thomas Hofmann, Thomas Gruber, Benjamin Schöne

**Affiliations:** 1 Experimental Psychology I, Institute of Psychology, Osnabrück University, Lise-Meitner-Straße 3, 49076 Osnabrück, Germany; 2 Industrial Design, Engineering and Computer Science, University of Applied Sciences Osnabrück, Osnabrück, Germany; 3 Department of Psychology, Norwegian University of Science and Technology, Trondheim, Norway

**Keywords:** Virtual reality, Reality, 2D, Visual processing, Event-related potential

## Abstract

Characteristics of real-life objects, such as binocular depth, potentially yield visual processes going beyond what examinations of planar pictures as experimental cues can reveal. While virtual reality (VR) is used to approximate real-life features in experimental settings, this approach fundamentally hinges on whether the distinct modalities are processed in a similar way. To examine which stages of early visual processing depend on modality-specific characteristics, our study compares the electrophysiological responses to 2D (PC), VR, and real-life (RL) objects. To this end, participants passively explored abstract objects in one of these modalities followed by active exploration in a delayed matching-to-sample-task. Our results indicate that all modalities fundamentally yield comparable visual processes. Remarkably, our RL setup evoked the P1-N1-P2 complex corresponding to the well-established ERP morphology. However, the magnitude of the ERP response during real-life visual processing was more comparable to the response to VR than to PC. Indicating effects of stereoscopy on the earliest processing stages, the P1 differentiated only between PC and RL, and the N1 differentiated PC from both other conditions. In contrast, the P2 distinguished VR from both other conditions, which potentially indicated stereoscopic visual fatigue. Complementary analysis of the alpha-band response revealed higher attentional demands in response to PC and VR compared with RL, ruling out that the ERP-based results are exclusively driven by attentional effects. Whereas comparable fundamental processes are likely occurring under all modalities, our study advises the use of VR if the processes’ magnitude is of relevance, emphasizing its value to approximate real-life visual processing.

**Supplementary Information:**

The online version contains supplementary material available at 10.3758/s13415-025-01294-0.

## Introduction

Although there are fundamental similarities between real-life objects and their representations in laboratory studies, real-life objects have unique characteristics that cannot be fully captured by planar images, e.g., life size, stereoscopy, and depth (Marini et al., 2019; Nastase et al., 2020; Shamay-Tsoory & Mendelsohn, 2019; Snow & Culham, 2021). These perceptual characteristics arrange objects of different modalities along a realness continuum, with the highest degree of realness characterized by the graspability and actability of objects, promoting intentions to interact with them (Snow & Culham, 2021). Consequently, real-life objects mark the upper limit of this realness continuum, whereas simplified stimuli reach respectively lower degrees of realness depending on their actual equivalence to real-life features (Schöne et al., 2023; Snow & Culham, 2021; Wrzus et al., 2024).

The characteristics that differentiate real-life objects from their low-dimensional proxies potentially yield visual processes that go beyond what can be examined by using planar pictures as experimental cues (Marini et al., 2019). Importantly, potential differences in early visual processing might modulate higher cognitive processes, for example by affecting the formation of the percept (Kiefer et al., 2011). Particularly the early sensory response to stimulus presentation is known to be highly sensitive to diverse stimulus features, such as stimulus size and eccentricity (Busch et al., 2004), as well as color (Hillyard & Münte, 1984) and complexity (Tarkiainen et al., 2002). This sensitivity is reflected in the dynamics of the early event-related potential (ERP) waveform, typically characterized by three consecutive peaks in response to visual stimulus presentation: the P1, the N1, and the P2 components (Luck, 2014; Lunghi et al., 2015). The P1 marks the first positive peak approximately 100 ms after stimulus onset measured at occipital sensors, and is sensitive to features like size (Busch et al., 2004), arousal (Vogel & Luck, 2000), and selective attention (Hillyard et al., 1998). Whereas the P1 is not sensitive to task-related information, the N1 is associated with the selection of relevant information and discriminative processing but also spatial attention (Hillyard et al., 1998).

The N1 is characterized as a negative deflection following the P1 and usually peaks between 120 to 180 ms after stimulus onset measured at posterior to parietal sensors (Clark et al., 1994). It is followed by a subsequent positive deflection around 200 ms at central to posterior sensors, the P2 component, which is sensitive to attention and beyond that, to the processing of more complex stimulus features, the identification of target features, and stimulus categorization (for a comprehensive characterization of the respective components see Clark et al., 1994; Hillyard et al., 1998; Luck, 2014; Luck & Kappenman, 2011; Michel & Murray, 2012).

In one regard, the ERP’s sensitivity to specific features but also cognitive states (e.g., attention; Hillyard et al., 1998) allows for examination of the dynamical modulations of neural processes under highly controlled conditions by implementing slight and precise variations of stimulus presentations. Conversely, this might mean that the realness of a stimulus might have immense effects on the processes reflected in these neural dynamics, questioning the equivalent processing of real-life objects and their planar representations (Marini et al., 2019; Snow & Culham, 2021).

However, the stimulus-driven modulations of ERPs have only seldomly been contrasted between 2D conditions and real-life conditions, which may reflect the high effort required to present real-life stimuli in a controlled experimental context (Marini et al., 2019; Romero & Snow, 2019). As an exception, Marini et al. (2019) revealed a more negative deflection of the visually evoked potential to real-life objects compared to their matched photographs in the time window from 99 to 183 ms at occipital to posterior electrodes. Hence approximately corresponding to the P1-N1-P2 complex, this finding indicates a modulation of the early visually evoked response dependent on the presentation modality and was attributed to the stereoscopic features in real-life objects, i.e., the lack of stereoscopic disparity in their 2D representations.

Whereas these results imply a limitation in the transferability of processes identified in desktop-based (PC) experiments to real-life processes, a steady increase of Virtual Reality (VR)-based experiments proposes to bridge this gap, and thus to foster the transferability of experimental findings beyond the specific experimental context (for review, see de la Rosa & Breidt, 2018; Kothgassner & Felnhofer, 2020; Pan & Hamilton, 2018; Parsons, 2015; Parsons et al., 2020). The trend toward using VR to achieve higher realness compared with desktop-based settings is based on accumulating differences in cognitive processes assessed in traditional PC-based paradigms and VR (Bilgin et al., 2019; Dan & Reiner, 2017; Johnsdorf et al., 2023; Kisker et al., 2021; Schöne et al., 2021) or similarities between VR experiences and their real-life equivalents (Chirico & Gaggioli, 2019; Gorini et al., 2010; Schöne et al., 2023). Like real-life environments, immersive VR experiences offer a sensory-rich, surrounding context (Slater & Wilbur, 1997). On the perceptual level, immersive VR can be designed in a way that the stimuli within the virtual environment mimic realistic physical features, for example by offering binocular cues (Parsons, 2015; Wrzus et al., 2024). Building on these perceptual characteristics, interactive scenarios and stimuli approximate real-life interactions by responding to the user’s head and body movements, albeit usually omitting haptic sensations (Kisker, Lange, et al., 2021; Rizzo & Koenig, 2017; Tromp et al., 2020; Wrzus et al., 2024). Hence VR is placed between desktop-based settings and real-life settings along the realness continuum (Schöne et al., 2023), with the effective position between the two poles depending on the specific implementation of said immersive features. A systematic classification of VR along the realness continuum would provide decisive insights into which experimental setting provides a closer approximation of real-life processes, and thus superior transferability of experimental results to the latter. However, the majority of comparisons of the early visual response evoked across modalities are based on stimuli presented on a desktop contrasted with 3D stimulus presentation either by means of stereoscopic screens (e.g., televisions) or by means of VR head-mounted displays (HMDs). Differences found between these conditions have predominantly been associated with the availability or lack of stereoscopic features, or depth information per se. Several studies report an increased P1 amplitude in response to 3D stimuli compared to 2D stimuli, attributed to the variance in depth information (Avarvand et al., 2017; Oliver et al., 2018; Omoto et al., 2010). The N1 (Oliver et al., 2018; Pegna et al., 2018) and P2 (Omoto et al., 2010; Pegna et al., 2018) are similarly sensitive to stereoscopic information, responding with a higher amplitude to 3D materials. Opposing aforementioned results, other studies report lower P1 amplitudes to 3D stimuli compared with 2D stimuli (Johnsdorf et al., 2023) or do not find amplitude difference regarding either component between conditions varying in stereoscopic features (P1, N1, P2: Kalantari et al., 2021; P2: Oliver et al., 2018).

Whereas stereoscopy is frequently proposed to drive the observed amplitude modulations (Omoto et al., 2010; Pegna et al., 2018), the temporal dynamics of early visual perception, i.e., the respective components’ peak latencies, are relatively similar between PC and 3D conditions (Aksoy et al., 2021; Kalantari et al., 2021; Omoto et al., 2010; Pegna et al., 2018). Only few studies indicate otherwise, reporting earlier peak latencies for VR- compared with PC-based presentation (Sagehorn et al., 2024a). However, modulations beyond those of stereoscopy might contribute to the differences found. For example, attentional processes modulate the early visually evoked potential (Hillyard et al., 1998). Studies indicating modality-dependent differences in the magnitude of attentional processing when comparing VR-based and PC-based stimulus presentation (Kweon et al., 2018; Li et al., 2020; Schubring et al., 2020) are opposed by initial evidence proposing comparable attentional processing (Sagehorn et al., 2024b).

Most importantly, early visual processing of planar or virtual 3D objects has rarely been compared with real-life objects (for exceptions see Kisker et al., 2024a; Marini et al., 2019), leaving the extent to which early visual processing differs as a function of modality unresolved. However, differences in these early processes might shed light on upstream consequences. For example, a companion paper demonstrated that cognitive load during visual processing of real-life objects compared with the response to virtual 3D objects, while it differed significantly from the response to 2D images (Kisker et al., 2024a). Reflected in the induced theta-band response as a marker for nonphase-locked, higher cognitive processes, the differences found might result from early perceptual processes. Yet the analysis of the phase-locked, i.e., evoked frequency response as a marker for stimulus-driven processes drew an inconclusive picture. The evoked theta-band response differentiated 2D and 3D materials to some, yet not to the expected degree, leaving the question of how early, stimulus-driven visual processes compare between the three modalities unresolved.

To gain more precise insights into the early visual processes, the analysis of the visually evoked potential would provide insights into stimulus-driven modulations of the neural response, and into the timing and magnitude of potential modality-specific differences in visual processing. Although the canonical ERP components have been reported under diverse conditions, no study has rigorously contrasted the P1-N1-P2 complex between PC, VR, and real-life settings. This comparison would allow precise conclusions to be drawn about which stages of early visual processing are modulated by modality-specific sensory information. Conversely, it specifies the degree to which early sensory stimulus processing can be generalized both qualitatively and quantitatively across modalities, and which experimental settings exhibit processes approximating real-life processes more closely.

To explore differences in visual processing depending on modality, we conducted a comparative study realizing three matched conditions along the realness continuum: a desktop-based 2D setting (PC); an immersive, three-dimensional VR setting; and a real-life setting (RL) by means of a physical replication of the experimental setup. Participants were asked to perform a delayed matching-to-sample task, i.e., they passively explored abstract objects in either modality followed by an active exploration of the objects. While the PC and RL conditions each represented opposite poles on the realness continuum, the VR condition was placed between these two poles. It differed from the PC condition in particular because of the three-dimensional presentation, as well as the graspability and actability of the stimuli implemented via hand-tracking in VR. On the basis of these immersive features, the VR condition was intended to approximate the RL condition, although the latter was further characterized by the haptics of the stimuli.

We compared the morphology of early visual processing: i.e., the latencies and amplitudes of the canonical P1-N1-P2 complex, between the three experimental conditions to specify the stages of early visual processing at which it is modulated by modality-dependent characteristics. Based on previous literature, we expected to obtain the canonical P1-N1-P2 complex under all conditions. Yet previous literature provides little evidence that the temporal dynamics of visual processing reflected in the peak latencies of the individual components differ between modalities. However, we performed a peak latency analysis before the amplitude-based analyses to account for rarely found latency effects (Sagehorn et al., 2024a, 2024b).

Because the majority of studies comparing the visually evoked potential to 2D and 3D stimuli indicate higher amplitude levels in response to divergent 3D materials, we expected the P1-N1-P2 complex to differentiate between PC-based stimulus presentation and both VR-based and real-life presentations. We hypothesized that no significant difference would be found between the responses to the latter two. However, previous studies found amplitude modulations beyond effects of stereoscopy: e.g., related to attentional processing (Kweon et al., 2018; Li et al., 2020). Accordingly, the P1-N1-P2 complex might differentiate between objects presented in immersive VR or the real world. To examine the potential effects of attentional processing during stimulus presentation, the alpha-band response (ABR; 8–13 Hz; Berger, 1929) was taken into account. We focused on the nonphase-locked (induced) portion of the frequency response which offers insights into cognitive processes not precisely time-locked to stimulus onset (Eckhorn et al., 1990). Specifically, the induced ABR (iABR) inversely relates to cortical activity. A decrease of the iABR measured at occipital sensors thus indicates an increase of activity in visual areas (Feige et al., 2005) and is associated with visual (Clayton et al., 2018) and attentional processing (Klimesch et al., 1997). Hence, in case the iABR corresponds to the potential ERP differences between conditions, these differences can be attributed to attentional processing rather than, for example, mere stimulus-dependent features.

## Methods

The current results originate from a large-scale dataset using the same experimental procedure and data acquisition (for preregistration see: https://osf.io/e64cd/?view_only=cba2d368d21f483a85dc701c0b11d216). The analyses at hand are restricted to a subset of this dataset relevant for the present research question. Companion publications will address other subsets of this comprehensive dataset (e.g., Kisker et al., 2024a). Because all publications are based on the same experiment, we aimed for highest congruence of the in-depth methodological description, resulting in overlaps between the publications, concerning e.g., figure details depicting the experimental procedure and the methodological details.

### A priori power analysis

G*Power (Faul et al., 2007) was used to estimate the sample size by calculating an a-priori power analysis. The expected effect size was estimated at the lower bound of a large effect, i.e., *η*^*2*^ = 0.14 (Cohen, 1988), based on large effects reported in preceding studies contrasting the electrophysiological correlates of perceptual and mnemonic processes under distinct modalities (Johnsdorf et al., 2023; Schöne et al., 2023). The power analysis was based on an ANOVA as statistical analysis, a power of 0.95, and an α error probability of 0.05, because the estimated sample size had to be suitable for divergent analyses planned for different data subsets of the experiment. A required total sample size of 98 participants was estimated by G*Power. We aimed for 99 participants, i.e., 33 participants per condition to achieve equal sample sizes.

### Participants

The study was approved by the local ethics committee (reference: Ethik 5/2023) and conducted in accordance with the declaration of Helsinki. All participants gave informed written consent before participation.

We recruited 107 participants via the psychology student email list, the University’s online bulletin board, and by students of the bachelor’s and master’s degree program of psychology. Participants were screened by means of a short interview and self-report (anamnesis) for psychological, psychiatric or neurological disorders, and substance intake. Three participants were excluded from participation, because they did not meet the inclusion criteria. Four participants had to be excluded owing to technical issues during data acquisition, and one participant refrained from participation. All participants had the chance to win one of two 50€ vouchers, and received partial course credits or 20€ after participation.

The data of 99 participants (31 males, 68 females, none intersex) were included into analyses (PC: *n* = 33 [60.6% female], *M*_Age_ = 22.48, *SD*_Age_ = 3.06; VR: *n* = 33 [69.7% female], *M*_Age_ = 22.21, *SD*_Age_ = 3.80; RL: *n* = 33 [75.8% female], *M*_Age_ = 22.76, *SD*_Age_ = 2.82).

### Stimulus material

**Fig. 1:**
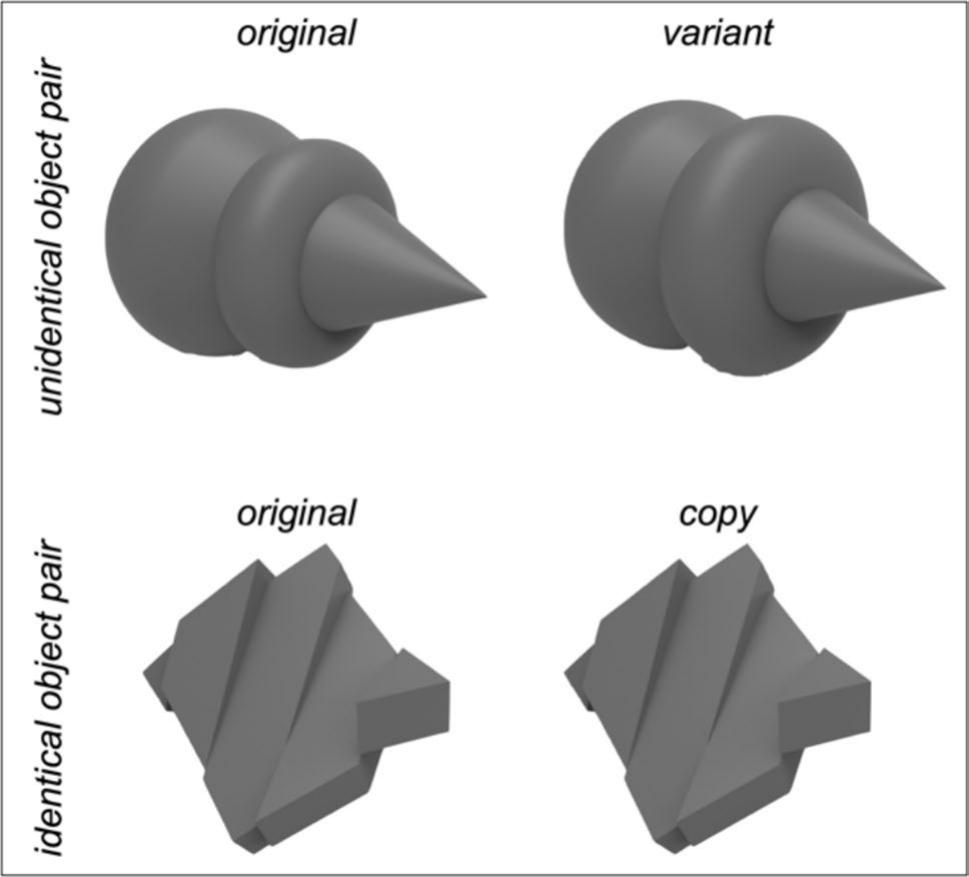
Exemplary stimulus material. *Note*. The stimulus material of the encoding session included 80 object pairs. Unidentical object pairs consisted of an original object and its slightly variated version (upper pair; the size of the right-most ring-shaped element of the object was varied). Identical pairs consisted of an original object and its copy (lower pair). The pairs of objects were presented as 3D printed objects in reality, virtual 3D objects in VR, and 2D objects on a screen. Participants archived an average accuracy of 65% in discriminating whether the object pairs were identical or not, where chance was 50% (see 2.5 Procedure for a detailed description of the task). Please refer to Kisker et al. (2024a) for the analysis of the behavioral data

A total of 160 abstract 3D objects were modeled using Supershapes (version 0.0.3, https://andrewmarsh.com/software/supershapes-web/) and Rhino (version 5; Robert McNeel & Associates, Seattle, WA). Each object was counterchecked for potential semantic associations by two investigators. If at least one investigator associated an object with any semantic label (e.g., a fruit, a spinning top, etc.), this object was excluded and replaced with a newly modeled object. Eighty objects were used during the encoding session (see below). To create pairs of objects, half of these objects were remodeled with a slight variation, thereby creating two marginally different versions of this object (original and variant, i.e., unidentical object pairs; Fig. 1). For the remaining 40 objects, a copy of the original was created (original and copy, i.e., identical object pairs; Fig. 1). All object pairs were printed using a 3D printer for presentation in the RL condition. These models were as well presented in 2D on a conventional monitor for the PC condition and in 3D using a VR-HMD for the VR condition. The original versions from the identical pairs were printed twice. This ensured that participants would not be able to determine whether the object pairs were identical or unidentical solely by taking individual characteristics of the texture into account which resulted from the printing process. The physical texture of the 3D prints was photographed and applied to the virtual objects in both the PC and the VR condition. The maximum vertical viewing angle on the objects was calculated to be 7.01° based on the largest object in the RL condition (10 cm^3^) and a seated distance of 65 cm between object and viewer. The viewing angle of the other two conditions was matched to the RL condition by taking the same object as in the RL condition as a reference and scaling it in the VR environment and on the screen to 10 cm^3^ as well. All further objects were scaled in reference to it, while maintaining a distance of 65 cm between the viewer and the objects in each condition. The remaining 80 original objects were only used during the retrieval session and presented as 2D renderings of the objects, i.e., planar pictures.

### Setup

The experiment comprised an encoding session and a retrieval session. Both were carried out on the same day and included EEG acquisitions. A delayed matching-to-sample paradigm was conducted in the encoding session (for details see below). The retrieval session was based on a remember/know recognition memory task. The latter will be outlined only briefly in this publication, because the analyses at hand exclusively focus on the encoding session. The data obtained from the retrieval session are addressed in a companion publication focusing on a separate research objective.

The encoding session was either carried out as a real-life (RL) condition, using a regular PC monitor (PC condition), or using a VR-HMD (VR condition). All conditions were implemented following the same concept to maintain high comparability. Participants were randomly assigned to these conditions, yet participants wearing glasses were randomly assigned to the RL or the PC condition due to technical constraints. The choice of a between-design was essential for the recognition memory task, allowing for examination of potential differences in memory performance between these conditions. Additionally, we considered the duration that would have resulted from a within-design—three times 60 min encoding plus the retrieval task—to be unreasonably long for participants, which might have caused fatigue or drop-outs.

**Fig. 2:**
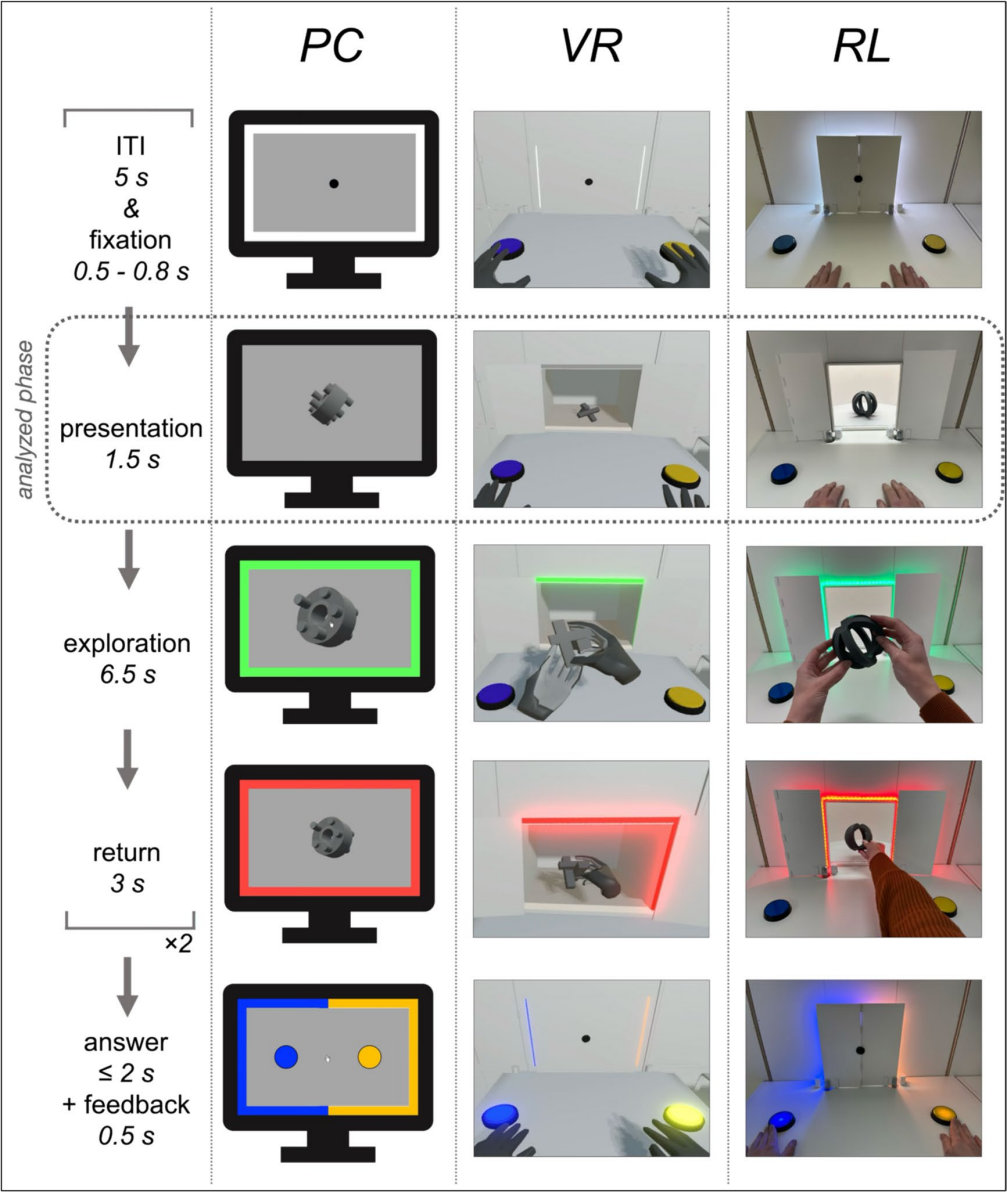
Experimental procedure of the encoding session per experimental condition. *Note.* Participants performed a delayed matching-to-sample task either in a conventional laboratory condition (PC), in Virtual Reality (VR) or in a real-life condition (RL). The task’s sequence is illustrated schematically and was identical across conditions. The virtual representation of the hands in the VR condition corresponded to the position and movements of the participants physical hands which were tracked by a LEAP motion controller. The grey dotted line marks the phase of the task on which the research question and analyses at hand are based. Please note that the VR condition is not depicted true to original brightness in this figure since a screenshot of the test leaders control view was used for illustrative purposes. This figure resembles the illustration of the experimental procedure of the companion papers to achieve highest congruence and transparency between publications (Kisker et al., 2024a)

All participants wore earplugs to reduce external noise. Participants of the RL condition sat at a table that held two mechanical buttons as an input module for the participants’ responses to their task during encoding (see Procedure and Fig. 2). They faced the back of a shelf at a distance of 65 cm (see Fig. 2). A window in the shelf’s back was covered by two doors. The doors were connected to a stepper motor and controlled by a microcontroller (Raspberry Pi4; Raspberry Pi Ltd, Cambridge, GB). Their movement resulted in a sound of approximately 80 dB. Around the edges of the window, a light strip with 90 LEDs was attached to provide a color-coded light signal. A black fixation point was attached to the doors. The program controlling the doors and the LED strip was developed in Thonny (https://thonny.org; version 3.3.14) using Python (version 3.9.2).

The PC and the VR condition were implemented in Unity (version 2020.3.3f1; Unity Technologies, San Francisco, CA). Both were aligned to the RL condition. For the VR condition, an HTC Vive Pro 2 HMD (HTC, Taoyuan, Taiwan; 2448 × 2448 pixels per eye, 60° field of view, 90-Hz frame rate) was used. The laboratory environment and experimental setup of the RL condition was recreated (Fig. 2). In particular, the mechanical buttons, the LED strip, and the participants’ surroundings and seating position corresponded to those in the RL condition. The doors’ sound was recorded from the RL condition and played at 80 dB via the integrated headphones of the VR-HMD. The hand tracking device *Leap Motion Controller 1* (Ultraleap Limited, Bristol, England) was used to enable real-time interactions with the objects corresponding to the RL condition. Hence, the VR-HMD conveyed stereoscopic cues, while the *Leap Motion controller* allowed for grasping and interacting with the objects within the virtual environment. The hardware did not generate any haptic feedback when grasping the objects in VR.

The PC condition was designed to maintain key features of conventional screen-based designs in order to replicate well-established findings on the one hand, and to cover for the lower pole of the hypothetical realness continuum on the other hand. Consequently, the objects were presented two-dimensionally on a regular monitor (24″, 1920 × 1200 pixels resolution, 60-Hz frame rate) against a light grey background. The monitor was placed on top of a table equivalent to the one used in the RL condition and in front of the very same shelf to match the laboratory context of both other conditions. A conventional computer mouse was used to interact with the objects. To match the viewing angle of both other conditions, participants were seated at 65 cm distance to the screen. Two 2D buttons appeared on the screen whenever responses were required. Hence, they were only visible during the answer phase and feedback (Fig. 2). A colored frame was displayed along the edges of the screen to mimic the LED strip (Fig. 2). Doors in the same color as the background were used to match the visual impression of the doors’ opening and closing process the RL and VR conditions. The doors’ sound was played using regular speakers at 80 dB positioned on both sides of the monitor.

*Retrieval session.* The retrieval session was conducted subsequently to the encoding session. Because the data obtained from the retrieval session will be examined in a companion paper, it is outlined only briefly for the sake of completeness. Participants were led into another laboratory to perform a remember/know recognition memory task on a conventional monitor (24″, 1920 × 1200 pixels resolution, frame rate of 60 Hz). The task comprised 160 experimental trials. Planar pictures of the 80 objects from the encoding session as well as 80 new objects were displayed in randomized order. Participants had to indicate for each original object whether they remembered the object from the encoding session or not while EEG data were acquisitioned. Only the original objects, i.e., none of the copies and variants of each pair, were used for the recognition task.

### Procedure

After completing the anamnesis, the EEG was set up and the participants were either equipped with the VR-HMD, seated in front of the 2D monitor (PC condition), or seated at the real table (RL condition). They were instructed to perform a delayed matching-to-sample task, i.e., two objects of a pair (Fig. 1) were presented consecutively with a short time delay during which the original object had to be retained. After the offset of the second object, participants indicated whether the pair of objects was identical or not. The experiment started with a training session consisting of two trials, one with an identical object pair, and one with an unidentical object pair. This way, participants were familiarized with the procedure and the handling of the respective condition (e.g., interacting with the object via the hand tracking device). The objects presented during training were not used in the experimental trials. The training session could be repeated as often as required. After ensuring that the participants understood their task and were able to perform it without technical hurdles, the experimental trials were started.

For each of the 80 trials, the color-signal provided information about the current phase of the trial: Each trial started with a white signal (5 s). Its offset indicated the fixation phase (randomly 0.5–0.8 s). Participants in the RL and the VR condition were to fixate the dot attached to the closed doors, while a fixation dot appeared in the center of the screen in the PC condition (see Fig. 2: fixation). The doors opened (0.15 s to fully opened) and the participants looked at the object presented in a random rotation (1.5 s; see Fig. 2: presentation). The start of the exploration phase was indicated by the onset of a green signal (6.5 s). In the RL condition, participants reached for the 3D-printed object and explored it using their hands. Interaction with the virtual 3D object in VR was realized by real-time hand tracking (see Fig. 2: exploration). In the PC condition, participants used a standard mouse to explore the 2D version of the object. They were able to zoom it in and rotate it around all axes. After the exploration time, the color-signal started to flash red (3 s). The objects had to be returned in the RL and the VR condition, and to be clicked with the computer mouse in the PC condition to zoom them out (see Fig. 2: return). After the doors closed (0.65 s to fully closed), the color-signal turned white (5 s), indicating the inter-trial-interval (ITI). The object was exchanged by either their copy or their variant, and presented following the same procedure. After the doors closed and covered the second object, the color-signal turned blue and yellow, the buttons in the RL and VR condition lit up and the buttons appeared on the screen in the PC condition. Participants had to indicate whether the two explored objects were identical or not within two seconds (see Fig. 2: answer). The button assignment was counterbalanced between participants, i.e., whether the blue button had to be pressed for identical object pairs and the yellow button for unidentical object pairs or vice versa (50% of participants each). If participants answered incorrectly or too slowly, the color-signal turned off (0.5 s). In case of a correct answer, the color-signal flashed blue and yellow for half a second instead. After 40 trials, the experiment was paused for two minutes to give participants a break with the possibility to extend it if needed. Exemplary video material of the three encoding modalities can be found online in the subfolder “*Exemplary video recordings of the encoding modalities*” at OSF (https://osf.io/6trmu/?view_only=6229545683e540609783fcc3ad862a0a).

### EEG Recording

A total of 128 active electrodes by BioSemi (Amsterdam, Netherlands) were attached in accordance with the international 10–20-system. Additionally, a Common Mode Sense (CMS) and a Driven Right Leg (DRL) electrode were used as ground and reference electrodes. An electrooculogram (EOG) was obtained using four electrodes attached around the eyes. The data were recorded at a sampling rate of 512 Hz and an online-filtered at 0.016–100 Hz. During the encoding session, LabRecorder was used to record the EEG data stream and synchronize it with the triggers send by Lab Streaming Layer (LSL by SCCN, https://github.com/sccn/labstreaminglayer). The latency of the event triggers sent via LSL was counterchecked and corrected using a photodiode. During the retrieval session, EEG was recorded using ActiView702 (BioSemi, Amsterdam, Netherlands).

### EEG Preprocessing

The EEG data analyzed in this publication are exclusively based on the phase of the delayed matching-to-sample task during which the objects were presented statically and were only to be looked at by the participant (see Fig. 2: illustration). During this time window, participants were instructed not to move or blink in order to avoid eye- and motion-induced artifacts. The EEG data were preprocessed using Matlab (version R2023a, MathWorks Inc.) and EEGLAB (version 2023.0, Delorme & Makeig, 2004). As a first step, bad channels were identified using Artifact Subspace Reconstruction (ASR; default settings; Mullen et al., 2015). On average, 0.74 (*SD* = 2.12) channels were interpolated (*M*_RL_ = 0.46, *M*_VR_ = 1.52, *M*_PC_ = 0.27). The EOG was excluded for all further preprocessing steps. Trials including errors (e.g., if an object was not properly displayed) were marked during the encoding session by the investigators and excluded from analyses. This applied to only ten datasets, for which an average of 1.8 trials were excluded (*M*_RL_ = 0.21, *M*_VR_ = 0.33, *M*_PC_ = 0). The data were epochized from −500 ms before the door started opening to 1500 ms afterwards. The average reference was calculated and applied to all electrodes. Channels deviating by more than two standard deviations from the mean of all electrodes were identified and interpolated (*M* = 5.39, *SD* = 1.82; *M*_RL_ = 5.76, *M*_VR_ = 5.73, *M*_PC_ = 4.70). A FIR band pass filter from 0.25 Hz to 30 Hz and linear detrending were applied. An independent component analysis (ICA) was used to detect and remove artifacts. Components classified as eye (0.8 probability), muscle, heart, channel noise and line noise (0.9 probability each) were marked for exclusion using the IClabel function (Pion-Tonachini et al., 2019). On average, 1.63 (*SD* = 1.26; *M*_RL_ = 1.94, *M*_VR_ = 1.42, *M*_PC_ = 1.52) components were excluded. A baseline of −500 to −300 ms before the doors started opening was applied.

**Fig. 3:**
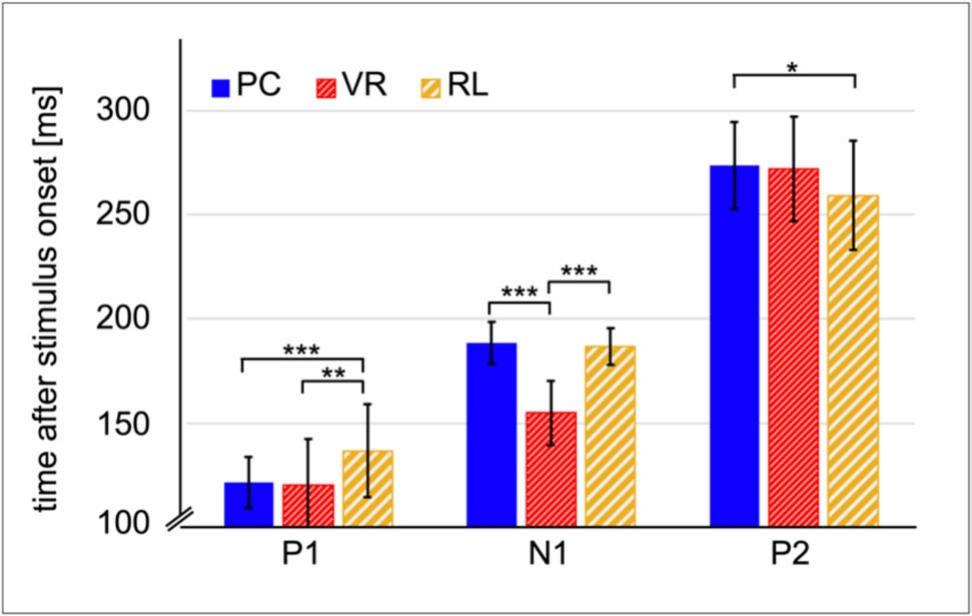
Peak latency after stimulus onset per ERP component and condition. *Note*. The error bars depict the standard deviation from the conditions mean. Significant comparisons are marked with *p < .017 (Bonferroni-corrected threshold), **p < .01, ***p < .001

**Fig. 4:**
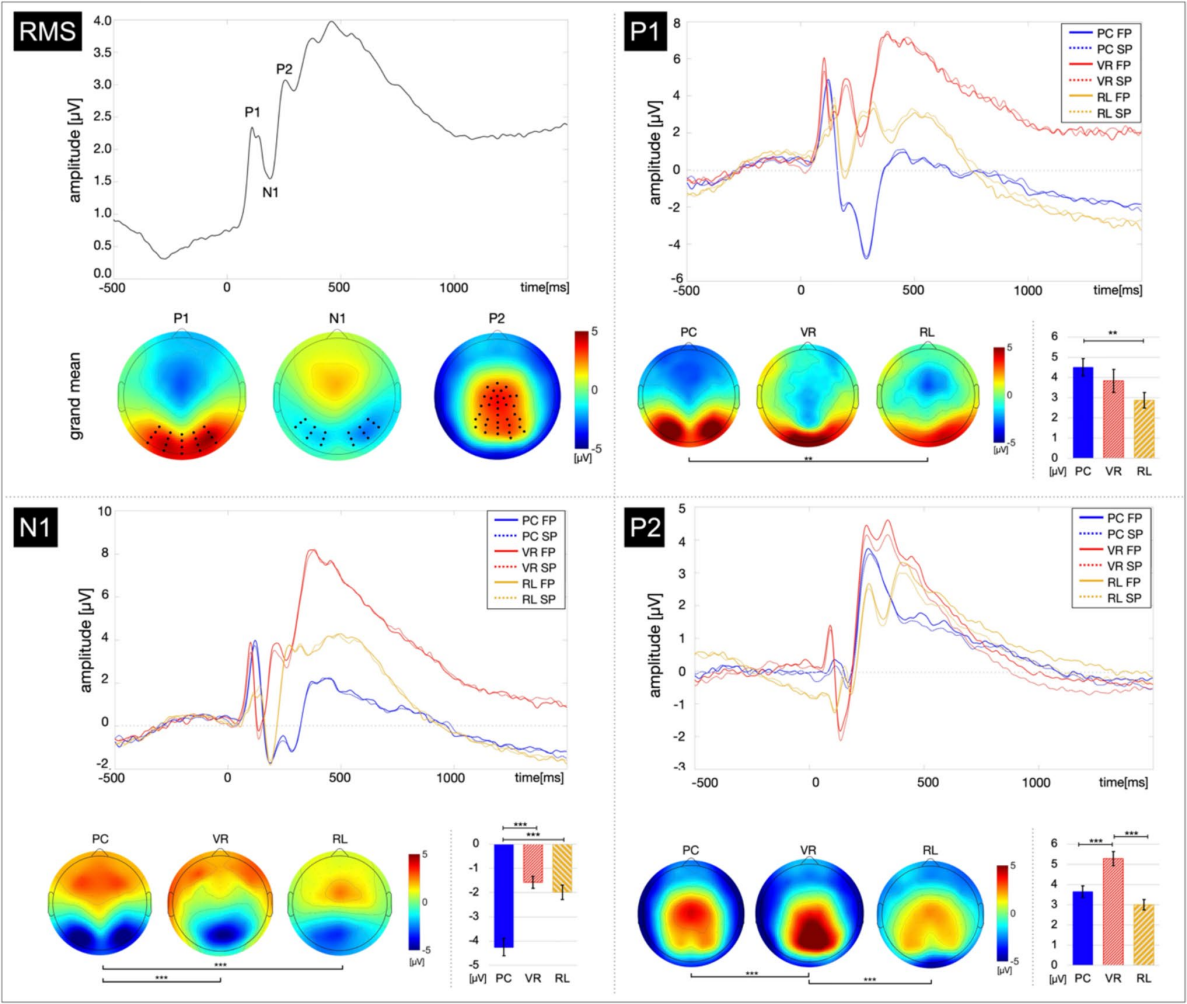
Root-mean square across conditions, line plot, and topographical amplitude distribution per ERP component and condition. *Note*. (RMS): The topographies are averaged across conditions and presentation times, resulting in the grand mean per ERP component. The bold black dots in these topographies mark the electrodes chosen for analyses. The line plot depicts the RMS averaged across all electrodes. (P1, N1, P2): The line plots depict the time course of the amplitude distribution averaged across the electrodes chosen for analyses. The respective topographies depict the peak amplitude distribution per condition. The bar plots depict the peak amplitude per condition, and their error bars indicate the standard error of the mean. The line plots were not rescaled with regards to the latency analyses while the topographies and bar plots were corrected by means of the peak-to-peak analyses. Significant comparisons are marked with *p < .017 (Bonferroni-corrected threshold), **p < .01, ***p < .001

**Event-related potentials**. Grand averages were calculated per combination of condition (PC, VR, RL) and presentation (first presentation, second presentation) for the analyses of the event-related potential (ERP). The root mean square was calculated averaged across all electrodes and conditions, and used to identify the P1, N1, and P2 peak latencies. These latencies were used to plot the amplitude distribution averaged across conditions as topographies (peak latency ± 10 ms; see Figs. 3 & 4, RMS). The electrodes used for further analyses were chosen based on regions of interest (ROI) derived from previous literature, and adapted by means of visual inspection of the regional means within the ROIs in these mean topographies (see Fig. 4, RMS). The identified electrode clusters were used to perform a latency analysis per component, i.e., the peak latency was determined for each specific condition and ERP component. The individual peak latency per condition and component was further used for comparison of the amplitude levels.

For the analyses of the amplitude levels, the peak amplitude level was calculated as the average across the amplitude at the peak latency ± 15 ms. Hence, the peak amplitude level was compared between conditions while controlling for (potentially) divergent peak latencies.

The amplitude levels were analyzed by means of peak to peak analyses. The P1 as the first ERP component after stimulus onset is based on the baseline level for each condition. In contrast, the amplitude of the N1 and P2 components depend to some degree on the absolute amplitude level of the respective preceding component (see Fig. 4, lineplots). Hence, to ensure that potential differences in the N1 and P2 components between conditions were not solely based on absolute amplitude differences resulting from the respective preceding component, the mean P1 amplitude level was subtracted from the mean N1 amplitude level for the comparison of the N1 between conditions, and the N1 amplitude level was subtracted from the P2 amplitude level for the comparison of the P2 between conditions.

**Fig. 5:**
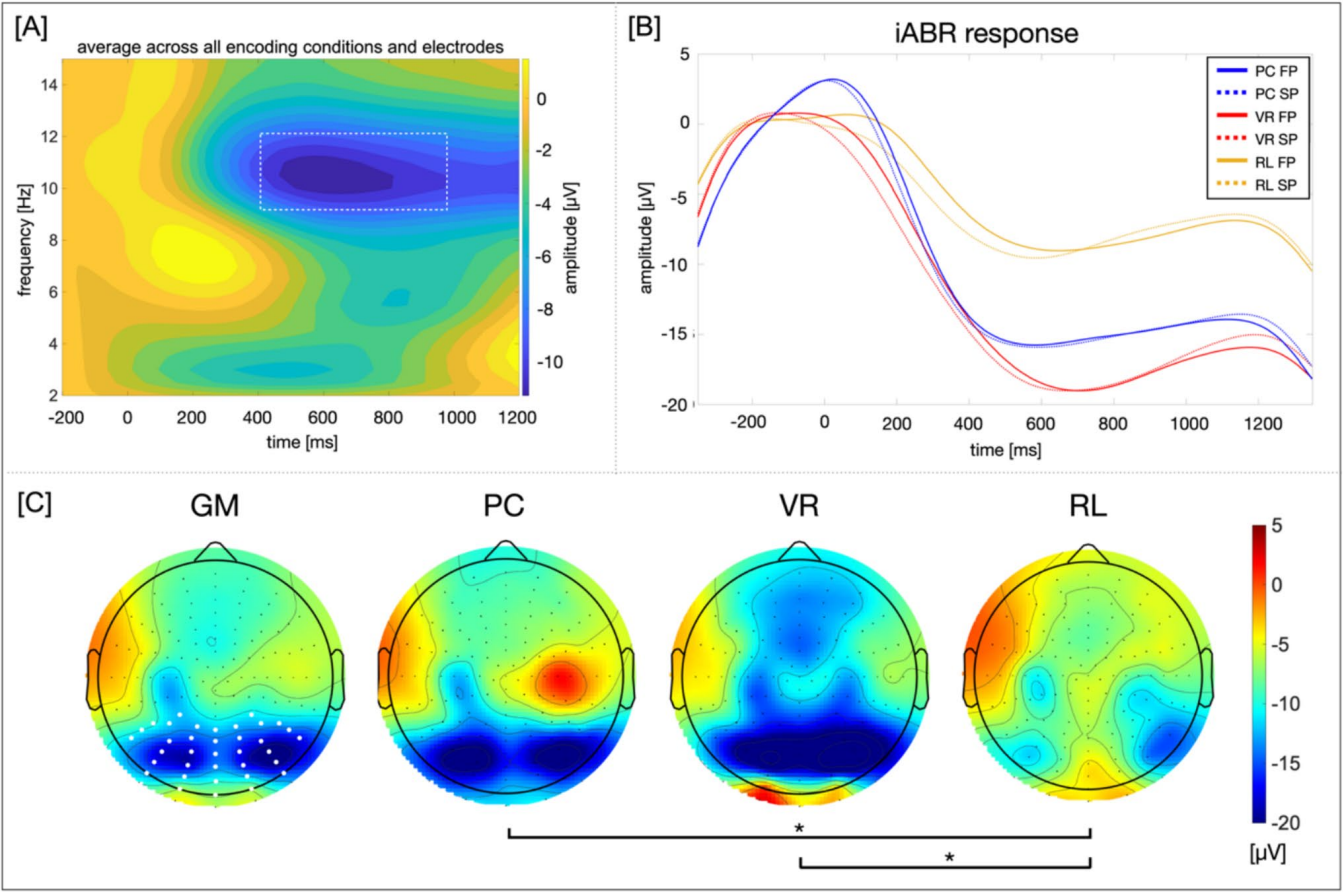
Induced alpha-band response (iABR) after stimulus onset. *Note*. (A) The white rectangle marks the frequency range and time window included into analyses. (C) The GM topography results from averaging across conditions and presentation times. All further topographies depict the amplitude distribution per condition in the time window from 410 to 970 ms after stimulus onset. The bold white dots in the GM topography mark the electrodes included into analyses. Significant comparisons are marked with *p < .017 (Bonferroni-corrected threshold), **p < .01, ***p < .001

**Frequency domain**. Additionally, a Morlet wavelet analysis was conducted (see Bertrand & Pantev, 1994; Cohen, 2014) applying a cycle width of twelve cycles per wavelet and a frequency resolution of 0.5 Hz. These settings resulted in in 199 wavelets between 1 and 100 Hz. To allow for analysis of the non-phase-locked components, the time–frequency (TF) amplitudes were averaged across single-trial frequency transformations. Otherwise, the induced activity tends to cancel out if trials are averaged due to a jitter in latency of the induced oscillatory activity after stimulus onset (Eckhorn et al., 1990). Hence, the ERP was subtracted from the induced response per trial before conducting the frequency decomposition. This procedure ensures that the analyses of induced activities is independent of the phase-locked processes reflected in the evoked oscillatory activity (Busch et al., 2006; Gruber et al., 2004; Kisker et al., 2024b for a similar procedure). To analyze the iABR, the specific frequency ranges and electrodes of interest were derived as regions of interest from previous literature. Both were further adapted by visual inspection of the data’s magnitude varying over time, i.e., the TF-plot, and the mean topography averaged across all conditions. Consequently, the frequency range from 9.5 to 12 Hz and a time window from 410 to 970 ms was chosen for analyses. A cluster of posterior electrodes including Pz, POz, Oz, O1, O2, and P1-P10 and 14 neighboring electrodes was included in the analyses (marked in Fig. 5).

### Statistical Analyses

The ERP peak amplitudes and latencies were both analyzed using a mixed 3 × 3 × 2 ANOVA including the within-factors COMPONENT (P1, N1, P2) and PRESENTATION (first presentation, second presentation), and the between-factor CONDITION (PC, VR, RL). The iABR was analyzed using a mixed 2 × 3 ANOVA including the within-factor PRESENTATION (first presentation, second presentation) and the between-factor CONDITION (PC, VR, RL). In case the *Mauchly*-Test indicated a violation of sphericity, the Greenhouse–Geisser correction was applied. Significant main effects or interactions were followed by *t*-tests for independent samples which were corrected for inequality of variances if indicated by the *Levene*-test. Because the same main effect or interaction was always the basis of three post-hoc tests, the alpha level was Bonferroni-corrected with α = 0.05/3 = 0.017. The respective effect sizes, *η*^*2*^ for the mixed ANOVA and Cohen’s d for *t*-tests, were calculated. Inferential statistics were complemented by calculating the Bayes Factor (*BF*_10_) for each *post-hoc t*-test to allow for more robust conclusions on differences between conditions and, more importantly, potential similarities between conditions. The* BF*_10_ for independent samples was calculated using JASP (JASP Team, 2024). A *BF*_10_ > 1 favors the H1, while a *BF*_10_ < 1 favors the H0.

## Results

### ERP latency

﻿The mixed ANOVA indicated significant main effects for the factors COMPONENT (*F*(1.82, 174.26) = 1570.56, *p* < 0.001, *η2* = 0.94) and CONDITION (*F*(2, 96) = 10.43, *p* < 0.001, *η2* = 0.18), whereas no main effect of PRESENTATION was found (*F*(1, 96) = 0.15, *p* = 0.902). Furthermore, a significant interaction of the factors COMPONENT and CONDITION (*F*(3.635, 174.26) = 18.24, *p* < 0.001, *η2* = 0.28) was found. No further interactions reached significance and the respective statistical reports can be found in supplementary material (S1). For the subsequent post-hoc *t*-tests, the data were averaged across the factor PRESENTATION.

Descriptively, the P1 occurred fastest after stimulus onset in the VR condition (*M* = 120.18 ms, *SD* = 22.20 ms) and the PC condition (*M* = 121.33 ms, *SD* = 12.31 ms). Its latency did not differ significantly between both conditions (*t*(64) = 0.26, *p* = 0.795, *BF*_10_ = 0.26). Yet, both differed significantly from the RL condition, which occurred last (*M* = 136.66 ms, *SD* = 22.38 ms; RL vs. VR: *t*(64) =  − 3.00, *p* = 0.004, *d* =  − 0.74, *BF*_10_ = 10.14; RL vs. PC: *t*(49.75) =  − 3.45, *p* < 0.001, *d* =  − 0.85, *BF*_10_ = 30.64).

The N1 occurred fastest in the VR condition (*M* = 154.88 ms, *SD* = 15.45 ms), second in the RL condition (*M* = 186.91 ms, *SD* = 8.95 ms), and last in the PC condition (*M* = 188.42 ms, *SD* = 10.13 ms; Fig. 3). The N1 latency of the VR condition differed significantly from both other conditions (VR vs. RL: *t*(51.31) =  − 10.30, *p* < 0.001, *d* =  − 2.55, *BF*_10_ > 1000; VR vs. PC: *t*(55.21) =  − 10.42, *p* < 0.001, *d* =  − 2.56, *BF*_10_ > 1000), whereas no significant difference was found between the PC and RL conditions (*t*(64) = 0.64,* p* = 0.52, *BF*_10_ = 0.30).

In contrast, significant differences in the P2 latency were found between the RL and the PC conditions (*t*(64) = 2.47, *p* = 0.016, *d* = 0.61, *BF*_10_ = 3.14). The difference between RL and VR did not reach significance after Bonferroni-correction, and the *BF*_10_ anecdotally favored the H1 (*t*(64) = 2.02, *p* = 0.047, *d* = 0.50, *BF*_10_ = 1.40). Moreover, the P2 latency did not differ between PC and VR (*t*(64) = 0.28, *p* = 0.784, *BF*_10_ = 0.26). Descriptively, the P2 peak occurred fastest in the RL condition (*M* = 259.14 ms, *SD* = 26.25 ms), followed by the VR condition (*M* = 271.96 ms, *SD* = 25.24 ms) and latest in the PC condition (*M* = 273.53 ms, *SD* = 20.85 ms).

As a consequence of the significant differences found between conditions concerning the peak latencies (Fig. 3), the analyses of the amplitude levels were corrected for these differences by considering the individual peak latency when calculating the mean peak amplitude per condition.

### ERP amplitudes

The mixed ANOVA indicated significant main effects for the factors COMPONENT (*F*(1.82, 174.42) = 285.14, *p* < 0.001, *η2* = 0.75) and CONDITION (*F*(2, 96) = 13.66, *p* < 0.001, *η2* = 0.22), whereas no main effect of PRESENTATION was found (*F*(1, 96) = 0.10, *p* > 0.751). Furthermore, a significant interaction of the factors COMPONENT and CONDITION (*F*(3.63, 174.42) = 9.08, *p* < 0.001, *η2* = 0.16). Consequently, the data were averaged across the factor PRESENTATION for the subsequent post-hoc *t*-tests. No further interactions reached significance. Please see supplementary material (S2) for the report of all nonsignificant interactions.

The amplitude of the P1 component was significantly more positive in the PC condition than in the RL condition (*t*(64) = 2.84, *p* = 0.006, *d* = 0.70, *BF*_10_ = 6.90). No differences were found between the PC and VR condition (*t*(64) = 0.93, *p* = 0.355, *BF*_10_ = 0.37), as well as between the RL and the VR condition (*t*(64) = 1.40, *p* = 0.165, *BF*_10_ = 0.58; Fig. 4).

The N1 component followed a similar trend: The amplitude was significantly more negative for the PC condition compared with the RL condition (*t*(64) =  − 4.89, *p* < 0.001, *d* =  − 1.02, *BF*_10_ > 1000) as well as compared with the VR condition (*t*(56.52) =  − 6.19, *p* < 0.001, *d* =  − 1.52, *BF*_10_ > 1000). The RL condition and the VR condition yielded no significant differences in the N1 amplitude (*t*(64) = 1.06, *p* = 0.293, *BF*_10_ = 0.41).

Regarding the P2 component, the amplitude differed between the PC and VR conditions (*t*(64) =  − 3.62, *p* < 0.001, *d* =  − 0.89, *BF*_10_ = 48.09), and between the RL and VR conditions (*t*(64) =  − 5.23, *p* < 0.001, *d* = 1.28, *BF*_10_ > 1000). In contrast, it did not differ between the PC and RL conditions (*t*(64) = 1.68, *p* = 0.098, *BF*_10_ = 0.83).

### Induced alpha-band response

The mixed ANOVA indicated significant main effects for the factor CONDITION (*F*(2, 96) = 3.42, *p* < 0.001, *η*^*2*^ = 0.07). Neither a main effect of PRESENTATION (*F*(1, 96) = 0.01, *p* > 0.05), nor an interaction of both factors (*F*(2, 96) < 0.001, *p* > 0.05) was found. The post-hoc *t*-tests were thus performed between conditions averaged across the factor PRESENTATION.

The iABR of the PC and VR conditions did not differ significantly (*t*(64) = 0.61, *p* = 0.542, *BF*_10_ = 0.30), while the RL condition exhibited a significantly less negative iABR compared with both other conditions (RL vs. VR: *t*(39.26) =  − 2.58, *p* = 0.014, *d* =  − 0.64, *BF*_10_ = 4.0; RL vs. PC: *t*(43.52) =  − 2.28, *p* = 0.014, *d* =  − 0.56, *BF*_10_ = 2.19). Descriptively, the VR condition exhibited the most negative going iABR (Fig. 5).

## Discussion

This study was designed to specify the stages of early visual processing at which it is modulated by modality-dependent characteristics, and thereby to unravel the extent to which visual processing varies or compares along a realness continuum. To this end, the canonical P1-N1-P2 complex was examined in response to objects presented on a conventional, two-dimensional desktop (PC condition), virtual 3D objects presented in immersive virtual reality using a head-mounted display (HMD; VR condition), and 3D-printed real-life objects (RL condition). The RL condition was set up as a physical replica of the conventional experimental setup, and recreated in VR.

Most importantly, our real-life setup evoked the canonical P1-N1-P2 complex comparable to both other presentation modalities and to the morphology well-established in the broad research background. Beyond the global resemblance of the visually evoked response, our results revealed local differences between the three presentation modalities as well. In detail, the ERP’s discriminatory power between conditions depended on the specific component. While the P1 amplitude only differentiated between the PC and the RL conditions, the N1 amplitude differentiated the PC condition from both other conditions. In contrast, the P2 amplitude distinguished the VR condition from both other conditions. In a similar vein, the component’s latencies indicated differences in processing speed, however, not in a unidirectional way. These findings are complemented by the iABR indicating higher cognitive demands under the PC and VR conditions compared to the RL condition, ruling out that the ERP-based findings can be explained exclusively by effects of attention.

Although previous findings did not necessarily give reason to expect differences in the temporal dynamics of early visual perception between modalities (Aksoy et al., 2021; Kalantari et al., 2021; Omoto et al., 2010; Pegna et al., 2018), our results reveal differences in processing speed. These differences cannot be attributed to technical artifacts which were carefully ruled out (see *Methods*). In particular, the P1 and N1 occurred fastest in the VR condition, immediately followed by the PC condition and, lastly, the RL condition. However, the P2 was observed in the RL condition first. Moreover, while the PC and VR conditions yielded comparable P1 and P2 latencies, they differed regarding the N1 latency. In a similar vein, the VR and RL condition differed in the P1 and N1 latency but not in the P2 latency. Consequently, our findings do not indicate a sustained processing speed advantage for any particular condition throughout the P1-N1-P2 complex.

An apparent explanation for the differences in the component’s latencies lies in the complexity of the stimuli by means of the availability of depth information. Depth information like shading and perspective, which are immediately available for 3D objects, potentially enhance processing speed (Sagehorn et al., 2024a, 2024b). However, this explanation implies that the latencies under VR and RL conditions would be more comparable to each other than both to the PC condition, which is not supported by our data. Moreover, the majority of previous studies comparing 2D and 3D materials revealed no latency effects based on stereoscopy (Aksoy et al., 2021; Kalantari et al., 2021; Omoto et al., 2010; Pegna et al., 2018). Hence, our results on peak latencies cannot be traced back to a binary categorization into two-dimensional and three-dimensional stimulus presentations.

Alternatively, attentional processing affects visual processing speed (Hillyard et al., 1998; Schuller & Rossion, 2001; Taylor, 2002). We found no difference between the iABR to VR and PC stimuli, whereas the desynchronization was significantly stronger for both conditions when compared to the RL condition. This oscillatory response potentially reflects more intense attentional processing under the PC and VR conditions compared with the RL condition. Because attentional effects are thought to enhance (Hillyard et al., 1998) and accelerate (Schuller & Rossion, 2001; Taylor, 2002) stimulus processing, this finding is in line with the earlier P1 peak in response to VR and PC conditions compared to the RL condition. Conversely, real, graspable objects have been discussed to draw higher attention than 2D or 3D images, as reflected in reaction time to stimulus-related tasks (Gomez et al., 2018). As our study allowed for interactions with objects under all conditions, the attentional resources were likely modulated beyond the interactivity of the stimuli. For example, the aforementioned study (Gomez et al., 2018) accessed attention during an interference-generating, stimulus-related task. This different task and its requirements might contribute to the reversed pattern of results. However, beyond the peak latency, the P1 amplitude is potentially indicative of attentional facilitation (Hillyard et al., 1998) or load (Fu et al., 2010) as well, being highest in the PC condition, followed by the VR condition, and finally by the RL condition. This interpretation is only coherent for the P1 component but does not reflect the differences found regarding both subsequent components. Our results indicate that it is essential to correct for peak latency when comparing the ERP across modalities.

When controlling for the differences in latencies, the overall morphology of the canonical ERP complex qualitatively corresponded between conditions, indicating rather quantitative differences. In particular, the ERP increasingly differentiated between conditions with each stage of visual processing in terms of magnitude reflected in the extent to which the amplitude increased or decreased, respectively. Regarding the P1, the VR condition formed the intermediate between both other conditions, thus, not significantly differing from these conditions, while the PC condition yielded a significantly stronger amplitude increase than the RL condition. At this stage of visual processing, we replicated the proposed differentiation between 2D and 3D stimuli by the P1 (Avarvand et al., 2017; Oliver et al., 2018; Omoto et al., 2010) only when comparing PC and RL. Overall, this suggests that the initial global impression of an object is processed relatively comparable across modalities in the first instance. Whereas the response to PC-based presentation significantly overestimated the P1 magnitude compared with real-life presentations, the P1 amplitude did not differentiate the VR condition from both other conditions. Hence, 3D objects presented in VR might probably share features with both. With respect to the RL condition, this might be binocularly mediated depth information, whereas VR and PC potentially overlap in monocularly mediated depth information. For example, Séverac Cauquil et al. (2006) report that the P1 is not sensitive to depth cues when it is varied between fully planar objects and an equivalent providing monocular depth cues.

Going one stage further, the N1 amplitude concisely differentiated between 2D and 3D objects: The PC condition yielded a significantly stronger amplitude decrease compared to both other conditions, underpinned by large effect sizes, whereas the RL and VR condition yielded no difference in amplitude decreases. This finding corresponds to previous studies proposing the N1 to be sensitive to stereoscopic information (Oliver et al., 2018; Pegna et al., 2018). Notably, these studies report a stronger amplitude to 3D materials compared with 2D materials, whereas our findings, vice versa, suggest a stronger amplitude decrease in response to 2D materials. Both of the aforementioned studies used abstract objects relatively comparable to our stimulus set, indicating that the differences do not result from semantical processing. Oliver et al. (2018) implemented a learning phase that facilitates the formation of a visual object representation. This representation largely depends on the visual input during encoding (Kiefer et al., 2011). Hence, downstream consequences resulting from retrieval processes might contribute to the observed differences in neural response to the objects as well. However, Pegna et al. (2018) applied a delayed matching-to-sample task comparable to our procedure, and likewise concluded that the N1 amplitude is stronger for 3D objects. Accordingly, the discrepancy in the results cannot solely be explained by the availability of an object representation. Alternatively, the mode of 3D presentation—i.e., the use of 3D glasses (Oliver et al., 2018; Pegna et al., 2018) versus an HMD and 3D printed objects—and the method of ERP analysis might contribute to the divergent direction of the N1’s sensitivity. While both aforementioned studies applied standard wave form analyses, we controlled for the absolute amplitude levels preceding each component of interest. While the direction of the effect might be partly determined by the approach used in the data analysis, the N1 nonetheless shows sufficient sensitivity in both cases to differentiate between 2D stimuli and different 3D presentation modes, i.e., reality, VR, and 3D glasses. Even more, the ERP potentially allows for earlier and more concise differentiation between 2D and 3D materials than the stimulus-locked frequency response (Kisker et al., 2024a). Although the evoked theta-band response has been discussed as a marker of stereoscopy (Tang et al., 2022), our companion publication showed that this differentiation is only partially accurate between 2D, virtual 3D, and real-life 3D. Within 300 ms of stimulus onset, the frequency response only differentiates between the desktop presentation and the real-life objects. Only in the later time window of 300–600 ms, a more precise differentiation between conditions on the basis of the evoked theta-band response was observed (Kisker et al., 2024a). The ERP is potentially more sensitive to stimulus-driven modulations and provides the incremental insight that visual processing differs earlier between conditions than would be estimated on the basis of frequency analysis.

Surprisingly, while the first two stages of visual processing revealed few significant differences in processing 3D objects presented in either VR or RL, the third stage of visual processing broke this trend. The P2 component differentiated the VR condition from both other conditions, yet did not differentiate between 2D presentation and real-life objects. Modulations of the P2 component are related, for example, to stimulus classification driven by top-down processes (Luck & Hillyard, 1994). Because the stimulus presentation was embedded in a delayed matching-to-sample task, cognitive processes facilitating the comparison of the original objects and their copy or variant might be at work at this stage. However, we did not observe differences between the neural responses to the original objects and their respective counterparts, rendering at least a significant involvement of discriminatory performance on the P2 modulations in this setup unlikely. Furthermore, the N1 is modulated by discriminative processes as well (Vogel & Luck, 2000); hence, a similar pattern would have been expected to emerge at this stage if the differences were predominantly driven by discriminatory performance.

A more technical approach to explain the observed differences, which might in particular account for differences between real-life 3D objects and virtual 3D objects, is stereoscopic visual fatigue. Stereoscopic visual fatigue relates to decreased performance of the visual system triggered by the eye’s accommodation to the disparity of two screens (Urvoy et al., 2013). In detail, stereo displays, such as HMDs, induce a vergence-accommodation conflict: While the eyes need to be focused on the display, 3D effects created by presenting differently shifted views to the eyes affords a vergence point, which mismatches the on-display focus (Harris et al., 2019; Urvoy et al., 2013). In our study, the vergence and accommodation cues were consistent for the PC and RL conditions but not the VR condition. This mismatch potentially limits visual performance and exemplifies limitations of VR displays related to fatigue, discomfort, and subjectively perceived size and distance (for in-depth discussions of stereo displays' limitations, see Banks et al., 2016; Harris et al., 2019; Rzepka et al., 2023). Previous studies related 3D visual fatigue to modulations of relatively late occurring event-related potentials (frontal P700, H.-C. O. Li et al., 2008; parieto-occipital P600, Mun et al., 2012). Albeit neither study directly corresponds to our experimental setup, they indicate that 3D visual fatigue likely affects ERP components occurring later than the P2 component. In contrast, Guo et al. (2022) demonstrated modulations of the posterior P2 by 3D depth perception and that increases in its amplitude and latency might link to stereo visual fatigue. With respect to these findings, our data showed a more positive deflection of the P2 component for the VR condition compared to both other conditions. This finding does not seem to stringently relate to 3D perception, because no significant difference was observed between the P2 evoked by planar and real-life objects. In combination with the iABR indicating more pronounced visual (Clayton et al., 2018) and attentional processing (Klimesch et al., 1997) in the VR condition (and PC condition) than in the RL condition, stereoscopic visual fatigue might account for the differences between visual processing of VR and real-life objects. However, the informative value of our study is limited, as we neither controlled for visual nor mental fatigue. Yet if the difference between VR and RL reflected in the P2 is related to visual fatigue, this discrepancy potentially can be minimized in the future through technological advances of VR systems.

Last but not least, it cannot be ruled out that the objects’ interactivity, or the kind of interaction affects visual processing. The generally possible interactions (i.e., bringing the object closer to the eyes, rotating it around all axes) were matched between conditions. This match was crucial, because the neural response is modulated by the perceived graspability of objects: When comparing real objects and their pictures, the difference in neural response is smaller when the real objects are not actually graspable due to a transparent barrier (Fairchild et al., 2021). However, while the generally possible actions were matched, the kind of interaction was more similar between VR and RL than both cases compared with PC. In this train of thought, VR and RL might exhibit cognitive processes related to the intention to grasp an object, i.e., motor planning, which would be minor for pictures (Fairchild et al., 2021; Snow & Culham, 2021). Fundamental research has shown that visual perception and motor preparation processes can generally overlap (Miller & Hackley, 1992), which raises the question of the extent to which these processes can mutually depend on each other. In particular, examinations of the lateralized readiness potential (LRP) occurring around 250 to 300 ms after stimulus onset indicate that visual information is transmitted to motor areas during early stages of stimulus processing (Miller & Hackley, 1992; Valt et al., 2017). Consequently, perceptual information relevant to motor planning are conveyed during these early stages to guide motor processes. Yet this does not imply that, vice versa, motor planning alters early perceptual processing at this stage. Albeit we cannot fully rule out effects of motor planning, we consider an effect of motor planning on the observed early visual response unlikely.

Ultimately, the overarching picture emerging from our results is that in particular the early stages of visual processing are relatively comparable across modalities by means of the overall ERP morphology. Most importantly, no significant differences were found between VR and RL concerning the P1 and N1 components, resulting in same theoretical interpretation derived from both. The differences found compared with the PC condition suggest early adaptations of visual processing to the specific modality. Moreover, the canonical ERP complex increased in sensitivity to modality-specific characteristics with subsequent stages of visual processing, e.g., reflected in the P2 component. Congruently, higher attentional load mirrored in a stronger iABR desynchronization in the VR and PC condition might contribute to partially greater amplitudes in response to both conditions compared with the RL condition. Because the objects of the real world correspond to the modality to which our brain has adapted evolutionarily (Johnsdorf et al., 2023; Ogmen et al., 2020; Snow & Culham, 2021), the weaker decrease of the iABR in the RL condition might indicate an inherent benefit reflecting lower cognitive demands for real-life objects during early visual processing. Remarkably, our companion paper indicated comparable cognitive load in response to virtual 3D (VR) and real-life objects, which was lower than the load in response to 2D objects. In line with this, Sagehorn et al., (2024a, 2024b) reported comparable stimulus-dependent attention, yet lower cognitive load for the perception of virtual 3D stimuli compared with their 2D equivalents. The differences in the attentional effects between the visual processing of virtual 3D objects and real objects are therefore not necessarily accompanied by differences in cognitive load.

## Limitations

Albeit we examined the earliest epoch of the procedure during which participants were asked to solely look at the presented object (see Fig. 2, presentation), it cannot be ruled out that higher processes linked to the delayed matching-to-sample task were already in progress. For example, processes might be initiated that contribute to the maintenance of the percept in working memory and promote discrimination performance. However, because we found no significant differences between the presentation of the original object and its variant or copy regarding any component, it is unlikely that these processes modulated the considered effects to a significant degree.

## Conclusions

Our results demonstrate that fundamental early visual processes are relatively comparable across modalities from a qualitative perspective but differ by quantitative means predominantly between planar 2D objects and both VR and real-life objects. Although previous studies gave little indication to presume so, our data indicate that peak latency must be controlled for with respect to the presentation modality and the respective ERP component to ensure a bias-free comparison of the amplitude levels across presentation modalities. Most importantly, the well-established ERP-morphology transferred to a real-life condition. The amplitude differences found were more of quantitative than of qualitative nature, indicating comparable fundamental processes along the realness continuum. However, the magnitude of the visual processing of real-life objects was more comparable to the neural response to VR than to desktop-based presentations. Particularly, the differences found compared with the PC condition suggest early adaptions of visual processing to the specific modality, which increases over time. In the light of the trend toward using VR to approximate real-life processes, our study advises the use of VR in particular if not only realistic processes per se but their magnitude is to be explored.

## Supplementary Information

Below is the link to the electronic supplementary material.

Supplementary file1 (PDF 271 KB)

## Data Availability

The datasets presented in this study can be found at OSF. All data covered in this publication is located in a subfolder entitled "*A comparison of the event-related potential to real-world and virtual reality and planar objects*" at OSF (https://osf.io/6trmu/?view_only=6229545683e540609783fcc3ad862a0a). Exemplary video material of the three encoding modalities can be found online in the subfolder “*Exemplary video recordings of the encoding modalities*” at OSF (https://osf.io/6trmu/?view_only=6229545683e540609783fcc3ad862a0a).

## Abbreviations

ABR: Alpha-band response
BF: Bayes Factor
EEG: Electroencephalography
EOG: Electrooculogram
HMD: Head-mounted display
iABR: Induced alpha-band response
ITI: Inter-trial-interval
PC: Personal computer
RL: Real-life
VR: Virtual reality
