# Supplementary material for: Comparative analysis of early visual processes across presentation modalities: The event-related potential evoked by real-life, virtual reality, and planar objects
Source: Cogn Affect Behav Neurosci. 2025 Apr 8;25(4):1022–39. doi: 10.3758/s13415-025-01294-0 (PMC12356721; doi:10.3758/s13415-025-01294-0)
Supplement: Supplementary file 1 — Supplementary file1 (PDF 271 KB) [file 13415_2025_1294_MOESM1_ESM.pdf]

Supplementary Material

S1. Mixed 3 x 3 x 2 ANOVA for the analysis of ERP peak latency including the within-factors COMPONENT (P1, N1, P2) and PRESENTATION (first presentation, second presentation), and the between-factor CONDITION (PC, VR, RL).

Tests of Within-Subjects Effects: Latency

Source		Type III Sum of Squares	df	Mean Square	F	Sig.	η^2
Components	Sphericity Assumed	2055469.950	2	1027734.975	1570.561	<.001	.942
	Greenhouse-Geisser	2055469.950	1.815	1132366.424	1570.561	<.001	.942
	Huynh-Feldt	2055469.950	1.887	1089414.466	1570.561	<.001	.942
	Lower-bound	2055469.950	1.000	2055469.950	1570.561	<.001	.942
Components * conditions	Sphericity Assumed	47750.402	4	11937.601	18.243	<.001	.275
	Greenhouse-Geisser	47750.402	3.630	13152.942	18.243	<.001	.275
	Huynh-Feldt	47750.402	3.774	12654.035	18.243	<.001	.275
	Lower-bound	47750.402	2.000	23875.201	18.243	<.001	.275
Error(components)	Sphericity Assumed	125639.867	192	654.374			
	Greenhouse-Geisser	125639.867	174.259	720.995			
	Huynh-Feldt	125639.867	181.130	693.647			
	Lower-bound	125639.867	96.000	1308.749			
Presentation	Sphericity Assumed	5.035	1	5.035	.015	.902	<.001
	Greenhouse-Geisser	5.035	1.000	5.035	.015	.902	<.001
	Huynh-Feldt	5.035	1.000	5.035	.015	.902	<.001
	Lower-bound	5.035	1.000	5.035	.015	.902	<.001
Presentation * conditions	Sphericity Assumed	742.916	2	371.458	1.120	.330	.023
	Greenhouse-Geisser	742.916	2.000	371.458	1.120	.330	.023
	Huynh-Feldt	742.916	2.000	371.458	1.120	.330	.023
	Lower-bound	742.916	2.000	371.458	1.120	.330	.023

Tests of Within-Subjects Effects: Latency

Source		Type III Sum of Squares	df	Mean Square	F	Sig.	η^2
Error(presentation)	Sphericity Assumed	31825.750	96	331.518			
	Greenhouse-Geisser	31825.750	96.000	331.518			
	Huynh-Feldt	31825.750	96.000	331.518			
	Lower-bound	31825.750	96.000	331.518			
Components * presentation	Sphericity Assumed	546.555	2	273.277	1.050	.352	.011
	Greenhouse-Geisser	546.555	1.790	305.294	1.050	.346	.011
	Huynh-Feldt	546.555	1.860	293.838	1.050	.348	.011
	Lower-bound	546.555	1.000	546.555	1.050	.308	.011
Components * presentation * conditions	Sphericity Assumed	2807.797	4	701.949	2.697	.032	.053
	Greenhouse-Geisser	2807.797	3.581	784.189	2.697	.038	.053
	Huynh-Feldt	2807.797	3.720	754.762	2.697	.036	.053
	Lower-bound	2807.797	2.000	1403.899	2.697	.073	.053
Error(components* presentation)	Sphericity Assumed	49971.301	192	260.267			
	Greenhouse-Geisser	49971.301	171.864	290.760			
	Huynh-Feldt	49971.301	178.565	279.849			
	Lower-bound	49971.301	96.000	520.534			

Tests of Between-Subjects Effects: Latency

Source	Type III Sum of Squares	df	Mean Square	F	Sig.	η^2
Intercept	21518932.021	1	21518932.021	23659.169	<.001	.996
conditions	18979.866	2	9489.933	10.434	<.001	.179
Error	87315.723	96	909.539			

S2. Mixed 3 x 3 x 2 ANOVA for the analysis of ERP peak amplitude including the within-factors COMPONENT (P1, N1, P2) and PRESENTATION (first presentation, second presentation), and the between-factor CONDITION (PC, VR, RL).

Tests of Within-Subjects Effects: Amplitude

Source		Type III Sum of Squares	df	Mean Square	F	Sig.	η^2
component	Sphericity Assumed	5515.960	2	2757.980	285.141	<.001	.748
	Greenhouse-Geisser	5515.960	1.817	3036.033	285.141	<.001	.748
	Huynh-Feldt	5515.960	1.889	2920.792	285.141	<.001	.748
	Lower-bound	5515.960	1.000	5515.960	285.141	<.001	.748
component * conditions	Sphericity Assumed	351.227	4	87.807	9.078	<.001	.159
	Greenhouse-Geisser	351.227	3.634	96.659	9.078	<.001	.159
	Huynh-Feldt	351.227	3.777	92.990	9.078	<.001	.159
	Lower-bound	351.227	2.000	175.613	9.078	<.001	.159
Error(compo-nent)	Sphericity Assumed	1857.086	192	9.672			
	Greenhouse-Geisser	1857.086	174.416	10.647			
	Huynh-Feldt	1857.086	181.297	10.243			
	Lower-bound	1857.086	96.000	19.345			
presentation	Sphericity Assumed	.218	1	.218	.102	.751	.001
	Greenhouse-Geisser	.218	1.000	.218	.102	.751	.001
	Huynh-Feldt	.218	1.000	.218	.102	.751	.001
	Lower-bound	.218	1.000	.218	.102	.751	.001
presentation * conditions	Sphericity Assumed	1.489	2	.745	.347	.707	.007
	Greenhouse-Geisser	1.489	2.000	.745	.347	.707	.007
	Huynh-Feldt	1.489	2.000	.745	.347	.707	.007
	Lower-bound	1.489	2.000	.745	.347	.707	.007
Error(presen-tation)	Sphericity Assumed	205.805	96	2.144			
	Greenhouse-Geisser	205.805	96.000	2.144			
	Huynh-Feldt	205.805	96.000	2.144			
	Lower-bound	205.805	96.000	2.144			

Tests of Within-Subjects Effects: Amplitude

Source		Type III Sum of Squares	df	Mean Square	F	Sig.	η^2
component * presentation	Sphericity Assumed	.604	2	.302	.134	.875	.001
	Greenhouse-Geisser	.604	1.780	.340	.134	.851	.001
	Huynh-Feldt	.604	1.849	.327	.134	.859	.001
	Lower-bound	.604	1.000	.604	.134	.715	.001
component * presentation * conditions	Sphericity Assumed	9.916	4	2.479	1.100	.358	.022
	Greenhouse-Geisser	9.916	3.560	2.785	1.100	.356	.022
	Huynh-Feldt	9.916	3.698	2.681	1.100	.356	.022
	Lower-bound	9.916	2.000	4.958	1.100	.337	.022
Error(compo-nent * presentation)	Sphericity Assumed	432.715	192	2.254			
	Greenhouse-Geisser	432.715	170.883	2.532			
	Huynh-Feldt	432.715	177.515	2.438			
	Lower-bound	432.715	96.000	4.507			

Tests of Between-Subjects Effects: Amplitude

Source	Type III Sum of Squares	df	Mean Square	F	Sig.	η^2
Intercept	1720.521	1	1720.521	239.257	<.001	.714
conditions	196.473	2	98.236	13.661	<.001	.222
Error	690.346	96	7.191			

S3. Mixed 2 x 3 ANOVA for the analysis of the induced alpha band response (iABR) including the within-factor PRESENTATION (first presentation, second presentation) and the between-factor CONDITION (PC, VR, RL).

Tests of Within-Subjects Effects: iABR

Source		Type III Sum of Squares	df	Mean Square	F	Sig.	η^2
presentation	Sphericity Assumed	1.305	1	1.305	.009	.925	<.001

Tests of Within-Subjects Effects: iABR

Source		Type III Sum of Squares	df	Mean Square	F	Sig.	η^2
	Greenhouse-Geisser	1.305	1.000	1.305	.009	.925	<.001
	Huynh-Feldt	1.305	1.000	1.305	.009	.925	<.001
	Lower-bound	1.305	1.000	1.305	.009	.925	<.001
presentation * condition	Sphericity Assumed	.034	2	.017	<.001	1.000	<.001
	Greenhouse-Geisser	.034	2.000	.017	<.001	1.000	<.001
	Huynh-Feldt	.034	2.000	.017	<.001	1.000	<.001
	Lower-bound	.034	2.000	.017	<.001	1.000	<.001
Error(presentation)	Sphericity Assumed	13992.142	96	145.751			
	Greenhouse-Geisser	13992.142	96.000	145.751			
	Huynh-Feldt	13992.142	96.000	145.751			
	Lower-bound	13992.142	96.000	145.751			

Tests of Between-Subjects Effects: iABR

Source	Type III Sum of Squares	df	Mean Square	F	Sig.	η^2
Intercept	38226.544	1	38226.544	83.989	<.001	.467
condition	3113.793	2	1556.897	3.421	.037	.067
Error	43693.004	96	455.135			
